# Application of the “echo-synch protocol” to advance pregnancy onset in ewe lambs at the first reproductive season

**DOI:** 10.3389/fvets.2023.1180857

**Published:** 2023-05-12

**Authors:** Francesca Daniela Sotgiu, Antonio Spezzigu, Cristian Porcu, Alberto Stanislao Atzori, Gian Simone Sechi, Valeria Pasciu, Giovanni Molle, Fiammetta Berlinguer

**Affiliations:** ^1^Department of Veterinary Medicine, University of Sassari, Sassari, Italy; ^2^Embryo Sardegna, Technology, Reproduction and Fertility, Sassari, Italy; ^3^Department of Agricultural Sciences, University of Sassari, Sassari, Italy; ^4^AGRIS Sardegna, Sassari, Italy

**Keywords:** ewe-lambs, ultrasonography, pregnancy, GnRH, profitability

## Abstract

**Introduction:**

This study assessed the efficacy and economic impact of a reproductive protocol based on repeated ultrasound scanning (US) associated with the use of GnRH to advance pregnancy onset in ewe lambs.

**Methods:**

Prepubertal ewe lambs (*n* = 133) were divided into three weight groups (High: HW *n* = 35; Medium: MW *n* = 65; Low: LW *n* = 33). Thereafter, animals were randomly allocated into two subgroups: GnRH, ewe lambs treated with GnRH analog and then exposed to rams; CTR, ewe lambs exposed to rams only. CTR groups were joined with rams as a single flock. GnRH groups were kept separate from rams receiving a single dose of gonadorelin (40 μg/head) and then were evaluated after a week of US. Animals showing corpora lutea received an injection of PGF2α analog (100 μg/head) and then were joined with rams. The remaining ewe lambs received a second dose of gonadorelin and were kept separate from the rams. After another week, animals were checked again and the ones showing corpora lutea were injected with the PGF2α analog, while the others received a third injection of gonadorelin. On the same day, all the animals were joined with rams. Pregnancies were confirmed within 30 days by US. The efficacy of the protocol was determined by assessing differences in the number of days required to achieve pregnancy rates of 25, 50, and 75% and in the total costs and incomes from birth to the end of first lactation within the groups.

**Results:**

The GnRH-MW group showed the best performances in reaching the threshold pregnancy rates of 25, 50, and 75%, but the effect of treatment was significant only at the 25% threshold (*p* < 0.01). Both low groups displayed an overall poorer performance at 50 and 75% thresholds than medium and high-weight groups (*p* = 0.01 and *p* < 0.01, respectively). The GnRH administration did not advance pregnancy onset in GnRH-HW compared with CTR-HW. In the balance between costs and income, the HW-CTR and MW-GnRH groups showed higher gross margins than the other groups.

**Conclusion:**

Using the US/GnRH protocol in ewe lambs appears technically and economically effective in animals that have not reached the optimal weight at the first breeding season, advancing ewe lambs’ pregnncies and increasing farm profitability.

## 1. Introduction

In Mediterranean countries, sheep production is an important economic, environmental, and social factor. Small ruminants have always had major importance in the Mediterranean basin, which is the only Region in the world where they raise approximately one-third of the global domestic ruminants, and this percentage has been stable since 1993 (1). The typical breeding system for dairy sheep such as that used in the Sarda breed implies one lambing per year, with the mating season starting in late spring for mature ewes and in late summer/early autumn for ewe lambs (approximately 20% of the flock) (2). Thus, the lambing period occurs between October and December for mature ewes, and between January and March for ewe lambs.

The reproductive management of dairy ewe lambs is based on the misconception that sheep puberty is mainly age-dependent, resulting in lambs being bred at more than 1 year of age (3), whereas it is well established that puberty can be reached when ewes reach between 60 and 65 percent of their mature live weight (4, 5). In practice, ewe lambs are often joined with males in a single flock without direct management and control of their reproductive activity.

This results in a low synchronization of pregnancy onsets and in a long lambing season with the bulk of the younger ewes lambing in March/April (pregnancy onset October/November) and having, therefore, a lower number of days in milk compared to adult ewes. This leads to a delay in the onset of animal productive activity and reduced economic income (5, 6). Moreover, ewe-lamb pregnancy rates are generally lower than those of adult ewes (2).

It has been reported that increasing the percentage of ewe lambs successfully bred at their first reproductive season is a means of increasing the flock’s productivity (7). Sheep lifetime productivity can be also extended by breading ewe lambs at an earlier age (8–11) since the number of lambing over a ewe’s lifetime is influenced by the age at first lambing and the frequency of pregnancies (12).

Breeding strategies for ewe lambs often imply the use of the ram effect and hormonal treatments (4). The ram effect is bonded to the interactions between sexual partners and it appears especially powerful to control reproduction (13). When used at the beginning of the breeding season it can advance the onset of puberty in ewe lambs by approximately 2 weeks (4, 14). However, other authors reported a poor response to the ram effect in ewe lambs (15).

In addition to conventional strategies, ruminants’ breeding plans can also imply the use of nutritional and hormonal treatments to enhance fertility rates (12, 16–19). In peri-pubertal lambs, the most common hormonal treatment used consists of an intravaginal progestagen-releasing device (IPRD) left *in situ* for 7 days, with or without eCG injection at device removal (4). The success of the treatment is more evident if applied in ewe lambs already exhibiting luteal activity (20). Moreover, Martinez et al. (12) found that the administration of a GnRH analog resulted in a higher proportion of prepubertal ewes ovulating when used in a protocol including IPRD, cloprostenol, and eCG, with a pregnancy rate similar to protocol with IPRD and eCG only. However, when mated at their first reproductive season, ewe lambs tend to have a lower response to synchronization protocols with respect to the adult ewes (12, 21–23).

The efficiency of reproductive management protocols can be increased by the use of ultrasonography (24). The main objectives of ultrasonographic investigation in ewes include visualization of the ovaries and uterine horns to determine whether or not there are pathological conditions of the tract, whether or not the animals are cyclic, and if they are, the phase of the cycle to perform pregnancy diagnosis and determine the stage to assess the growth and viability of the fetus. Overall flock reproductive management is enhanced by the real-time acquisition of information on ewes grazed or fed preferentially according to pregnancy status (25). In all aspects related to reproductive management, ultrasound also provides a connection between research and practice, as research results can be translated directly into the field (26).

Starting from these premises, the present study aimed at assessing the efficacy of a protocol based on ovarian ultrasound scanning and GnRH administration, compared to ram exposition only to advance pregnancy onset, narrow the lambing season, and increase the number of days in milk (DIM) in dairy ewe lambs entering their first reproductive season. The different outcome between protocols was evaluated by comparing the number of days from treatment start to pregnancy onset, DIM, protocol costs, and revenues at the end of the first lactation. Given the non-homogeneous live weights of this productive group, treatments were tested in different sub-groups having low, medium, and high live weights.

## 2. Materials and methods

**Figure 1:**
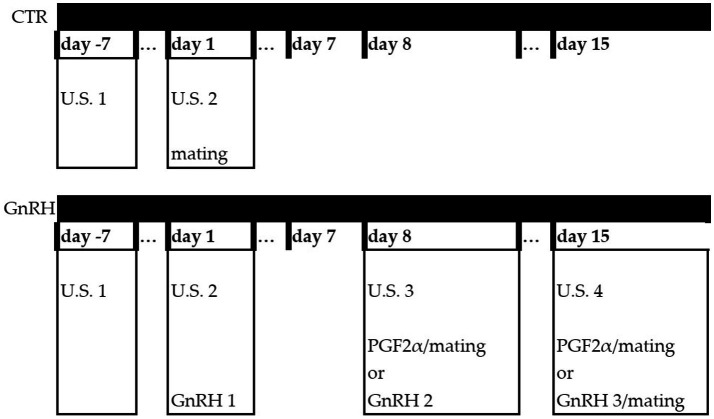
Experimental protocol—the figure shows the exact timing at which treatments and ultrasound scanning were performed.

The experiment was run from mid-July to the beginning of October 2019 at the Bonassai research station of Agris Sardegna (40° N, 8° E, and 32 m above sea level). Treatments were tested before the onset of the natural breeding season for the Sarda ewe lambs at this latitude (early autumn) (2). In brief (Figure 1), in mid-July, 133 prepubertal ewe lambs born in the previous autumn-winter lambing season, were selected from a single flock. During all experimental procedures, the animals were kept indoors in separate pens. Indoor feeding consisted of a unifeed mix of wrapped haylage of ryegrass and berseem clover, barley grain, whole peas, and soybean meal, supplemented with vitamins, minerals, and sodium bicarbonate. Ovarian status was evaluated by two consecutive ovarian ultrasound scans performed 8 days apart to ensure the absence of corpora lutea and, hence, of cyclicity. On the day of the second ovarian ultrasound scanning (US n 2, Day 1), ewe lambs were weighed before the morning meal by means of a digital scale for livestock. The animals were divided into three groups according to their live weight (live weight mean ± SE): high weight (HW), 39.1 ± 0.3 kg, *n* = 35; medium weight (MW), 33.8 ± 0.2 kg, *n* = 65; low weight (LW), 28.2 ± 0.3 kg, *n* = 33. Thereafter, within each weight group, animals were randomly allocated into two subgroups: GnRH, including ewe lambs treated with a GnRH analog (gonadoreline: Cystoreline^®^, Ceva Salute Animale, Italy) and then exposed to rams for mating; CTR, ewe lambs exposed to rams only as controls. During the trial, two ewe lambs were discarded from the CTR-MW and GnRH-LW groups because of acute trauma and diarrhea, respectively. Table 1 shows the animals grouping and body weight within each sub-group.

**Table 1:** Ewe lambs’ groups were matched by treatment (CRT; GnRH) and relative sub-groups according to body weight (HW, high weight; MW, medium weight; LW, low weight).

Group	Sub-group	*n*	Body weight Kg (mean ± SE)
CTR	HW	17	39.1 ± 0.4
	MW	31	33.8 ± 0.3
	LW	17	28.1 ± 0.4
GnRH	HW	18	39.2 ± 0.4
	MW	33	33.7 ± 0.3
	LW	15	28.2 ± 0.4

On Day 1, after group matching, CTR groups were joined with fertile rams fitted with crayon markers (here and thereafter rams/ewe lambs’ ratio was equal to 1/10) and managed as a single flock, whereas the GnRH groups were kept separated from rams and were administered a single dose of gonadorelin (40 μg/head, 0.8 mL). Following 1 week (Day 8), GnRH ewe lambs were evaluated by ovarian ultrasound scanning (US n 3). Those showing corpora lutea were injected with a PGF2α analog (cloprostenol, 100 μg/head; PGF Veyx, Vexy-Pharma GmbH, Germany) and then joined with crayon-marked fertile rams. Ewe lambs not showing signs of ovulation were injected with a second dose of GnRH. After another week (Day 15), they were checked again and the ones showing corpora lutea (US n 4) were injected with a PGF2α analog, while the remaining ones received a third and last injection of GnRH analog. At this point, all GnRH ewe lambs were joined with crayon-marked fertile rams. Ewe lambs’ mating behavior was checked daily for 120 days. The mated ewe lambs, once identified, were removed from the flock. After mating, pregnancy was then confirmed within 30 days using transrectal ultrasound scanning.

### 2.1. Ovarian ultrasound scanning

Ovarian ultrasound scanning was performed using a real-time, B-mode scanner (Aloka ProSound 2; Aloka Co. Ltd., Tokyo, Japan) fitted to a transrectal 7.5-MHz linear-array probe (82 mm prostate transducer UST-660-7.5, Aloka Co.), as previously described and validated (27). In each observation, the ovary was scanned several times from different angles to determine the presence and the number of corpora lutea. Pregnancy diagnosis was performed within 30 days post-mating using transrectal ultrasonography (Aloka ProSound 2, fitted to 82 mm prostate transducer UST-660-7.5, Aloka Co.). Pregnant sheep displayed enlargement of uterine horns and an embryo heartbeat was evident.

### 2.2. Flock management and performance

For each ewe, the days from the start of the experimental protocols to the conception were considered as days open. Ewes were kept in separate pens within the corresponding experimental group until pregnancy was confirmed and then added to the pregnancy group. The pregnancy length was considered equal to 150 days and the day of parturition was calculated as 150 days after the date of the last mating before pregnancy diagnosis. After pregnancy diagnosis, all the ewes were kept in a single group until lambing.

After lambing, all the ewes were kept in the same lactation group. In pregnancy and lactation, the ewes were fed the same diet used in the mating period and described before. In lactation, the animals were also fed with the same unifeed mix as a base, with the addition of the pasture, to which animals had limited time access depending on pasture availability. Ewes were milked twice a day with an automatic milking machine (DeLaval MidiLine SG300; DeLaval S.p.A. Via XXV Aprile, 220,097 San Donato Milanese (MI)) and received an individual amount of concentrate during milking (300 gr/head of a commercial pellet for lactating ewes).

Milk production per ewe was determined using the automatic records of the milking machine (software DeLaval Alpro S&G, Windows). The system implemented in the milking machine allowed us to gather daily records from each animal per milking and get the cumulative milk production from parturition until dry-off. The day of drying off was the same for all the ewes and set at 15th August, the date on which the farm actually stopped the seasonal milking. Some animals (18% out of the total of the experimental animals) had missing milking records because of a software failure; thus, their cumulative milk yield was assumed to be equal to the average value of the ewes from the same sub-group with similar lactation length.

### 2.3. The economic evaluation of the protocol’s effects

The economic evaluation of the performances included the evaluation of incomes and costs during the whole life cycle of the ewe lambs from birth to first dry-off in order to quantify differences related to the effect of the reproduction treatment. The evaluation accounted for rearing and reproduction costs sustained for each animal during the suckling and weaning phase *(phase I)*, the growing phase until Day 1 of the experimental protocol *(phase II)*, the days of the open phase from Day 1 of the experimental protocol until pregnancy onset *(phase III)*, pregnancy *(phase IV)*, and the first lactation from lambing to dry-off *(phase V)*.

The cost for *phase I*, i.e., for the ewe-lamb at weaning, was considered equal to 48.80 €, corresponding to the value of the lamb at 12 kg of body weight and 45 days of age. It was based on the estimation of the milk consumed by the lamb estimated from birth to weaning (28) and a milk value of 1.30 €/liter equal to the price of the milk sold as opportunity cost.

The feed cost of the growing phase, from weaning to the start of the experimental protocol *(phase II)*, was considered a function of the feed intake required to cover the maintenance and growth needs to reach the weight observed at the beginning of the trial for HW, MW, and LW. Data observed for the same breed in the same range of ages and weight were gathered by Ledda (29). The total feeding costs considered for *phase II* were, therefore, assumed to be 46.5 €, 40.71 €, and 27.98 € for HW, MW, and LW lambs, respectively, considering the growth patterns of the different weight ranges and the feed prices observed in the farm. For the growing phase, the diet consisted of a commercial pellet and ryegrass hay with a market price of 0.474 € per 0.135 €/kg, respectively. In the early-dry phase, the ewes were fed a commercial dry mix composed of ryegrass hay, barley grain, whole peas, and vitamin-mineral supplement having a cost of 0.27 €/kg of DM. The cost of *phase III*, from Day 1 of the experimental protocol until pregnancy onset, was calculated by multiplying the dietary feed cost of 0.26, 0.28, and 0.31€/d per head (based on the average cost of the farm diet provided in this period for LW, MW, and HW, respectively) and the number of days open.

The cost of gestation *(phase IV)* was obtained by considering feed costs of 0.34, 0.36, and 0.38 €/d of the pregnancy diet provided in the farm in the same period and the remaining days until lambing, calculated considering the feed consumption of each group based approximately on the dry matter intake of the ewes to cover maintenance and gestation requirements (30).

During *phase V*, for the lactating ewes, the dry matter intake from lambing to dry-off was individually estimated based on Pulina et al. (30) as kg/d of DMI per ewe = −0.545 + 0.095*animal live weight, kg^0.75 + 0.65*average milk, kg/d. Furthermore, the daily milk yield was calculated as total milk yield divided by the lactation length of each ewe. The total feeding cost in lactation was calculated by computing an average basis consumption of 1.58 kg/d of DM per head from unifeed intake (composed of haylage of mixed ryegrass and clover, barley grain, whole peas, soybean meal, vitamin-mineral supplement, and sodium bicarbonate; € 0.54/d) offered per group. Then, the difference between the estimated individual intake and the unifeed intake was assumed provided by the pasture intake (14.85 € per kg of DMI). The total feeding cost of lactation was then obtained by multiplying the lactation length and the daily cost calculated for each animal.

Relative costs per head were calculated for each treatment group. The cost of drug administration was calculated by multiplying the cost of the drug per mL used in the single dose (Table 2). For each ultrasound measurement, a unit cost of 1.00 € that included the veterinarian service was considered and multiplied by the number of ultrasound checks performed on each ewe.

**Table 2:** Drug costs and doses.

Drug (commercial)	Drug cost (€)	Drug cost (€/mL)	Drug dosages (mL)	Drug dose cost/head (€)
GnRH 100 mL	90.00	0.90	0.80	0.72
PGF 50 mL	46.00	0.92	0.40	0.37

Incomes were calculated by multiplying the liters of milk yield produced by each ewe by the milk price. The produced milk calculated was from the individual records of the milking machine. The price of the milk sold was set at 1.30 €/liter, considering the milk price at production paid by the local dairy plant for the same year.

The gross margin was equal to the income from milk over feed and reproduction cost from birth to first dry-off. Reproduction costs were applied considering the different protocols and individual treatments applied to each ewe.

Cost and income were calculated for each ewe and the average values were determined within groups to compare the effects of the experimental protocols on different weight groups.

In addition, a cost-share proportional to the number of empty ewes in each group was also assigned to each animal to account for the effects of the protocol on fertility and delayed pregnancies. The daily feeding cost of empty ewes was considered equal to that calculated in *phase III* of mating and applied for the experimental protocol duration. It is assumed that the inefficiency due to the reproduction protocols must be loaded on the economic performances of the lactating ewes of each treatment.

### 2.4. Statistical analyses

The efficacy of the protocols in advancing pregnancy onset was determined by assessing differences between groups in the number of days (from Day 1—treatment start—to pregnancy onset) needed to reach 25, 50, and 75% of pregnancy rates within each group (Table 3). Grubb’s test was used to detect outliers. Differences between groups on the number of days needed to reach 100% of the pregnancy rate were not taken into account because the threshold was not reached by all the groups. A general linear model was used to test differences in reproductive and economic evaluations considering the fixed effect of weight class (*n* = 3 levels; HW, MW, and LW) and treatment (*n* = 2 levels CTR and GnRH) and their interaction. Tukey test was preferred for comparisons. All results were expressed as mean ± SE and a probability of *p* < 0.05 was considered to be significant, whereas trends were considered when probability ranged between *p* = 0.05 and *p* = 0.1.

**Table 3:** Mean number of days from the start of treatment (Day 1) to the establishment of pregnancy rates of 25, 50, and 75% in ewe lambs of three different live weight (HW, MW, and LW), treated with exposure to rams or exposure to rams plus GnRH administration (CTR and GnRH).

	CTR-HW	CTR-MW	CTR-LW	GnRH-HW	GnRH-MW	GnRH-LW	HW	MW	LW	CTR	GnRH	SEM	Weight	Treat	*X*
25%	20.80^AB^	26.00^A^	23.60^A^	24.00^A^	14.00^C^	15.50^BC^	22.22	19.25	20.00	23.77^*^	16.71^*^	1.120	0.385	0.003	0.002
50%	22.78	28.69	34.67	23.67	20.12	35.13	23.22^b^	24.27^b^	34.88^a^	28.71	24.59	3.185	0.010	0.448	0.315
75%	30.15	40.29	49.46	32.14	29.48	50.33	31.19^b^	34.78^b^	49.88^a^	40.04	35.12	4.471	0.003	0.541	0.335

The upper case letters indicate the differences in the interaction between weights and treatments; the lowercase letters indicate the differences between weight groups, and asterisks (*) indicate the differences among treatments.

## 3. Results and discussion

### 3.1. Reproductive performances

It is well known that the attainment of correct body weight is part of the mechanisms that regulate the entry into puberty of lambs (31, 32). In this study, results showed differences in the timing of first pregnancy establishment between ewe lambs of different weights and subjected to different protocols (Table 3).

The association of US with GnRH treatment in ewe lambs exposed to rams was significantly effective in advancing pregnancy onset at the 25% threshold (*p* < 0.01). At this threshold, fewer days were needed to reach the first cutoff point for treated lambs than control ewe lambs in the low and medium-weight groups (Table 3, *p* < 0.01 for the interaction). On the other hand, at the 50 and 75% thresholds, differences in pregnancy onset were determined by the animal weight only (*p* = 0.01 and *p* < 0.01 as regard animal weight, respectively) rather than by the hormonal treatment or their interaction.

In lambs, physiological GnRH secretion increases during the transition from the pre-pubertal to the pubertal phase, stimulating the secretion of FSH and LH, and rams introduction lead to an increase in LH pulse frequency (33) and, hence, to follicular development (31, 34). The effect of the GnRH treatment was apparent only at the first threshold (25%), with a synchronizing effect able to advance the first pregnancy onset and narrow the lambing period in the low and medium-weight groups. Indeed, at the 25% cutoff, the time needed to conceive was 12 days shorter (i.e., – 46%) in GnRH-MW than the CTR-MW group and, as a result, the lambing period was narrowed. This difference, although not statistically significant, was observed also at 50 and 75% cutoffs (*p* > 0.05, Table 3). The reason for the fading effect of the hormonal treatment is unknown; however, the relatively small size of groups could be evoked as a probable reason for the lack of statistical evidence of the treatment effect.

In the other weight groups (LW and HW), the effect of GnRH treatment was not observed at 50 and 75% cutoff levels.

In the low-weight groups, ewe lambs were probably not ready for pregnancy due to their growth stage. After the first oestrus induced by the treatment, they possibly fell into a quiescent status, as already shown in anoestrus ewes by Laster and Glimp (35). This explains why moving from a 25 to 50% cutoff took 20 days in the GnRH-LW group (from 15 to 35 days) but only 11 days in the CTR-LW group (from 24 to 35 days, Table 3).

The poorer performances displayed by low-weight ewe lambs at 50 and 75% thresholds confirm that minimum weight is required for puberty onset (31, 32). These results prove that the low-weight group was unable to successfully respond to the protocol, suggesting that low-weight lambs should be managed with specific care in order to boost their weight gain to advance the puberty onset or to increase the probability to respond to a reproductive management protocol. In fact, besides the ram effect and hormonal protocols, nutrition planes are key factors that affect the onset of puberty for ewe lambs (10, 32, 36).

Finally, the lack of the effect of the hormonal treatment in high-weight groups (Table 3) may indicate that once the optimal weight is reached, choosing the ram exposure alone may be the most correct and also the cheapest choice. Indeed, the ram effect, when used at the beginning of the breeding season, is able to advance the onset of puberty in lambs by approximately 2 weeks (4, 14). This effect, just before the onset of puberty, can increase mean reproductive time and overall pregnancy rates (9, 37–39).

There were no statistical differences in the number of not lambed ewes between groups.

### 3.2. Production performances

Lactation performances were affected by pregnancy onset. Milk yield showed significant differences related to ewe-lamb’s body weight at mating, with higher production for HW and MW vs. LW groups (*p* < 0.001; Table 4). Milk yield also tended to be higher in GnRH than in CTR groups with a milk yield of 260 vs. 240 kg/yr. per ewe (*p* = 0.10; Table 4). Significant differences were observed for the daily yield (*p* < 0.05) and the lactation length (*p* < 0.01) per weight class (Table 4). Differences in milk production within weight class mainly depended on lactation length and were associated with the day of conception. Since the dry-off was equal for each animal, the earlier the conception the longer the lactation with higher cumulative production.

**Table 4:** Productive performances for the different reproduction treatments (CTR and GnRH) and weight groups (HW, MW, and LW).

	CTR-HW	CTR-MW	CTR-LW	GnRH-HW	GnRH-MW	GnRH-LW	HW	MW	LW	CTR	GnRH	SEM	Weight	Treat	*X*
Days open *(phase III)*	43.2	48.0	64.5	38.3	38.4	59.8	40.7^b^	42.9^b^	62.4^a^	51.2	43.1	2.63	0.04	0.23	0.89
Lactating ewes % by group	100%	90.3%	100%	94.4%	97%	93.3%	97.1%	93.8%	96.9%	95.4%	95.5%				
Lactation length, days	227	225	208	231	235	214	229^a^	230^a^	211^b^	221	229	2.54	0.006	0.18	0.85
Milk yield per lactation, kg/year per ewe	269	244	205	270	271	221	270^a^	258^a^	212^b^	240	260	4.83	0.001	0.10	0.45
Milk yield per lactation, kg/d per ewe	1.19	1.10	1.01	1.17	1.16	1.05	1.18^a^	1.13^a^	1.03^b^	1.10	1.14	0.021	0.026	0.52	0.73

Lowercase letters indicate the difference between weight groups.

From an economic point of view, the overall feeding cost of growing *phases I and II* was fixed at 95.30 €, 89.51 €, and 76.76 € for HW, MW, and LW, respectively. The cost of the reproduction protocol was 4.91 €/ewe on average for the GnRH groups, which was higher than for the CRT groups (2.00 €/ewe) as expected (Table 5). No significant differences were found between treatment and weight groups in the feed costs for *phase III* (Table 5). These costs were directly related to the days open following the treatment protocols. In low-weight lambs compared to the other groups, the feeding costs during the experimental period and until pregnancy onset were higher because a longer period was required to respond to the treatment.

**Table 5:** Feed and protocol costs for the different reproduction treatments (CTR and GnRH) and weight groups (HW, MW, and LW).

	CTR-HW	CTR-MW	CTR-LW	GnRH-HW	GnRH-MW	GnRH-LW	HW	MW	LW	CTR	GnRH	SEM	Weight	Treat	*X*
Protocol cost. €/ewe	2.00	2.00	2.00	4.86	4.87	5.08	3.50	3.49	3.48	2.00*	4.91*	0.143	0.68	0.001	0.68
Feed costs on days Open *(phase III)* €/ewe	24.57	26.38	27.81	22.32	21.55	27.50	23.41	23.89	27.66	26.3	23.1	0.80	0.25	0.21	0.97
Feed costs during pregnancy *(phase IV)* €/ewe	44.65	38.27	35.79	45.58	40.97	34.62	45.13	39.66	35.24	39.3	40.8	0.52	0.001	0.53	0.45
Rearing costs from birth to lambing. €/ewe *(phase I–IV)*	166.53	156.15	142.28	168.06	156.90	143.88	167.32	156.54	143.03	155.2	157.0	0.75	0.001	0.23	0.96
Feed cost in lactation *(phase V)*	136.44	132.64	120.64	138.40	140.36	125.33	137.45	136.62	122.84	130.4	136.5	1.15	0.24	0.05	0.91

Asterisks (*) indicate differences among treatments.

Feed cost during pregnancy *(phase IV)* and the total rearing cost from birth to first lambing *(phase I to IV)* were significantly different per class of body weight (*p* < 0.001), with decreasing values from HW to MW and LW but were not significantly affected by the treatment (Table 5). On average, the total rearing cost for a replacing lamb was equal to 155.20€ from birth to first lambing.

Milk incomes directly depended on the day of lactation and milk yield, as stated above, and like milk yield, they were significantly higher in higher weights groups HW and MW (*p* < 0.001) and tended to be higher for GnRH than for CRT groups (337.54 vs. 312.20, respectively; *p* = 0.1) (Table 6). The gross margin calculated as income over feed costs in lactation was significantly different per class of weight, which was lower for LW (153.10, €/ewe) than HW and MW (*p* < 0.001; Table 6), respectively, and it was equal to 199.20 and 212.34 €/ewe and not significantly different among them (Table 6). Similarly, numerical differences in gross margin from birth to first dry-off, including income from milk and costs for feed and reproduction, were +17.37 €/ewe higher in GnRH than in CRT. These values tended to be higher in the high and medium groups with respect to the lower groups (*p* = 0.065; Table 6). Values of gross margin from birth to first dry-off were numerically higher only in HW and MW and in the GnRH groups indicating that only animals from HW and MW categories were able to pay off the rearing and reproduction costs at the end of the first lactation (Table 6). The lambs that became pregnant earlier produced more milk as they had longer lactations.

**Table 6:** Incomes and net incomes from milk over feed cost for the different reproduction treatments (CTR and GnRH) and weight groups (HW, MW, and LW).

	CTR-HW	CTR-MW	CTR-LW	GnRH-HW	GnRH-MW	GnRH-LW	HW	MW	LW	CTR	GnRH	SEM	Weight	Treat	*X*
Incomes €/ewe	349.7^A^	317.2^A^	266.5^B^	351.0^A^	352.3^A^	287.3^B^	350.4^a^	335.9^a^	275.9^b^	312.2	337.5	4.11	0.001	0.10	0.44
Gross margin over feed cost in lactation, €/ewe	213.3^A^	184.6^AB^	145.9^B^	212.6^A^	211.9^A^	162.0^B^	212.9^a^	199.2^a^	153.1^b^	181.8	201.0	3.58	0.001	0.20	0.51
Gross margin from birth to first dry-off, €/ewe	46.7	28.4	3.6	44.5	55.0	18.1	45.6	42.6	10.1	26.6	44.0	3.44	0.065	0.30	0.50

The uppercase letters indicate the differences in the interaction between weights and treatments, and the lowercase letters indicate the differences between weight groups.

When the cost of empty ewes was considered to simulate a flock balance, the statistical analysis could not be executed, and the result was calculated per group and then expressed in €/yr. per ewe from birth to dry-off. The results reflected what was observed in lactating ewes. Furthermore, the differences among weight classes indicated that economic losses were higher in animals with lower weight at puberty and that ewes from MW seem to have the highest benefit from the protocol at the flock level (Table 7).

**Table 7:** Net income over feed and reproduction cost and milk losses, adjusted for empty ewes.

	CTR-HW	CTR-MW	CTR-LW	GnRH-HW	GnRH-MW	GnRH-LW	HW	MW	LW	CTR	GnRH	Weight	Treat	*X*
Empty ewes %	0.0%	9.7%	0%	5.6%	3.0%	6.7%	2.9%	6.2%	3.1%	4.6%	4.5%			
Protocol cost, €/empty ewe	–	2.00	–	5.81	5.81	6.16								
Feed costs €/empty ewe	–	293.40	–	105.10	97.80	85.30	52.55	222.26	189.08	293.40	288.20			
Feed and reproduction costs and milk losses, €/empty ewe	–	622.82	–	236.83	229.20	185.47	118.42	477.00	412.89	622.82	651.50			
Net losses in the flock, €/lactating ewe	–	22.24	–	13.93	7.16	13.25	7.16	14.46	6.21	10.61	10.55			
Net income adjusted, for empty ewes, €/milking ewe	4.91	−28.63	−20.26	−11.76	2.95	−25.13	−3.43	−11.79	−22.46	−17.14	−7.26	0.065	0.30	0.50

This result indicates that the use of hormones in the LW and HW animals may not be the most correct choice, but at the same time in LW, could limit losses compared with the use of the ram effect alone. On the other hand, it might be more convenient to focus on the feeding of low-weight ewe lambs before using hormones to achieve better reproductive responses by limiting the number of animals that conceive late in the season.

It is clear that proper growth of ewe lambs is essential for the attainment of puberty (40), and increasing the level of nutrition before the time of breeding can result in a higher proportion of ewe lambs successfully bred (41).

## 4. Conclusion

It is important to give more attention to ewe lambs through the focused use of ultrasonography, and when necessary, hormones because these tools can advance their pregnancy, improving the income of dairy sheep farms. Through ultrasound scanning, it was possible to identify the lambs that could receive GnRH treatment, and among them, animals with corpus luteum could be promptly joined to the rams by limiting the time spent in the days of the open phase. On the other hand, ultrasound scanning allowed the identification of ewe lambs that failed to respond to the hormonal treatment, supporting previous findings regarding the achievement of useful weight for the onset of reproductive activity. Thus, the targeted choice of lambs to be included in the breeding management protocol is the key point for obtaining a successful outcome even in economic terms.

The choice of animals to subject to hormonal treatment can point to lambs that are close to the optimal weight because they have been shown to repay the treatment costs by advancing pregnancy, narrowing the lambing season, and increasing the number of days in milk. The ewe lambs that had already reached the optimal weight effectively exploited the ram exposure alone, helping to limit the management costs of ewe lambs.

Regarding low-weight ewe lambs, they should be managed separately in order to ensure proper food access and consequently reach useful weight to successfully respond to reproductive management protocol, reducing feed costs. To this end, this study confirms the importance of measuring the live weight of ewe lambs since it has an overriding effect on dairy sheep reproduction and production career and, thus, has a great impact on the farm’s economic balance.

## Data availability statement

The raw data supporting the conclusions of this article will be made available by the authors, without undue reservation.

## Ethics statement

The animal study was reviewed and approved by Ethics Committee of AGRIS and the University of Sassari, in compliance with the European Union Directive 86/609/EC and the recommendation of the Commission of the European Communities 2007/526/EC. 2.1. The procedure was authorized by the Italian Ministry for Health (n. 705/2019-PR). Written informed consent was obtained from the owners for the participation of their animals in this study.

## Author contributions

FS, AS, CP, and FB designed the study. FS, AS, and CP performed the experiments. FS and CP prepared the manuscript. CP, AA, GS, GM, and FB analyzed the data. FS, VP, GM, and FB revised the manuscript. All authors contributed to the article and approved the submitted version.

## Funding

FS was supported by the Italian Ministry of Education, University and Research—PON ricerca innovazione 2014-2020 FSE REACT-EU azione IV.6 (contratti di ricerca su tematiche green) PON DM 1062MEDVET-IV6 CUP J85F21003600001. CP received a research grant funded by the Italian Ministry of Education, University and Research—PON ricerca innovazione 2014-2020 (asse 1 “capitale umano,” azione I.2 A.I.M.) grant code AIM 1887720-1. CUP J54I18000160001 and by the University of Sassari (Fondo di Ateneo per la ricerca 2020). The study was supported by the project e.INS—Ecosystem of Innovation for Next Generation Sardinia EC00000038, spoke 03—APPàre: smart and secure livestock farm APPlications to boost data-driven innovation along the food chain, funded by the European Program NextGenerationEU-Italy’s National Recovery and Resilience Plan.

## Conflict of interest

The authors declare that the research was conducted in the absence of any commercial or financial relationships that could be construed as a potential conflict of interest.

## Publisher’s note

All claims expressed in this article are solely those of the authors and do not necessarily represent those of their affiliated organizations, or those of the publisher, the editors and the reviewers. Any product that may be evaluated in this article, or claim that may be made by its manufacturer, is not guaranteed or endorsed by the publisher.
